# Racial Disparities and Black Parents’ School Preferences: Evidence from a Survey Experiment

**DOI:** 10.1007/s11256-025-00797-x

**Published:** 2026-01-03

**Authors:** Todd Hall, Chantal Hailey, Jeremy Prim, Janeria Easley

**Affiliations:** 1 University of Virginia, Charlottesville, VA USA; 2 University of Texas at Austin, Austin, TX USA; 3 University of California, Davis, Davis, CA USA; 4 Emory University, Atlanta, GA USA

**Keywords:** Black Parents, School Preferences, Racial Disparities, Suspensions, Test Scores, School Choice

## Abstract

Nearly half of Black parents have access to public school choice, but choice may not provide equitable and supportive school environments. Qualitative research documents Black parents worries that majority-White schools with more resources and higher test scores may over-discipline, underestimate, and exclude their children. Yet large-scale studies rarely examine what Black families desire in potential schools, including how they navigate potential tradeoffs between high school quality and denigrating school racial climates. This survey experiment examines the extent to which test score gaps and suspension gaps between Black students and their non-Black peers deter Black parents from choosing schools with higher overall test scores and lower overall suspension rates. We randomly assign a large, national sample of Black parents (N = 1,677) to examine a school profile vignette where the school has overall high academic achievement and low suspensions rates but includes either one, both, or neither academic and discipline gaps to assess how test score and suspension disparities affect Black parents’ school preferences and perceptions. We find that racial disparities in student discipline and academic outcomes, on average, diminish Black families’ desires to enroll in high-achievement schools and their perceptions of student belonging. These findings align with qualitative research showing that well-resourced, high-achieving schools are less appealing to Black families when they marginalize Black students and that schools’ unequal punishment of Black students shapes Black parents’ evaluations of potential educational spaces.

**Supplementary Information:**

The online version contains supplementary material available at https://doi.org/10.1007/s11256-025-00797-x.

Public school choice is increasingly common and is accessible to nearly half of Black parents (National Center for Education Statistics, 2021). Early proponents argued that publicly-funded school choice policies would reduce inequities by empowering low-income parents to access better schools (Chubb & Moe, 1990; Friedman & Friedman, 1980; Schneider et al., 1998). However, the merits of school choice for advancing educational equity have long been debated. Scholars demonstrate that school choice policies do not overcome racialized structural constraints nor systematic marginalization that Black students face in school. These policies often fall short of producing racial equity in access to higher performing schools and even exacerbate segregation (Cooper, 2005; Eisenlohr et al., 2023; Lareau et al., 2021; Saporito & Lareau, 1999; Simms & Talbert, 2019; Ukanwa et al., 2022). Moreover, Black families’ access to school choice policies may not guarantee access to equitable and supportive school environments. When choosing schools, Black parents worry that majority-White schools with more resources and higher test scores may over-discipline, underestimate, and exclude their children (Butler & Quarles, 2024; Lareau et al., 2021; Posey-Maddox, et al., 2021).

While a large quantitative literature documents White parents’ school preferences (Billingham & Hunt, 2016; Goldring & Hausman, 1999; Hailey, 2022; Holmes Erickson, 2017; Houston & Henig, 2021, 2022; Schneider & Buckley, 2002), large-scale studies rarely examine Black parents’ visions for schooling. This scant evidence prevents us from fully understanding what Black families desire in potential schools and how they react to information about various school contexts. Without hearing from Black parents directly, we cannot distinguish the effects of structural barriers preventing Black families from accessing higher-performing schools from expectations that such schools would be marginalizing for their Black children (Eisenlohr et al., 2023; see also Cooper, 2005; Posey-Maddox et al 2021; Lareau et al., 2021; Lewis-McCoy 2016). If concerns about racial inequities deter Black parents from schools, then promoting equitable enrollment in high-quality schools requires policymakers to address educational disparities in tandem with alleviating structural barriers.

The evidence on how Black parents navigate tradeoffs between potentially stigmatizing environments and school quality is limited and mixed. Some qualitative studies find that Black parents engage in constant “racialized risk assessment”, feeling torn between schools with overall higher student academic outcomes and racialized harm versus lower performing schools where they worry less about their Black children being singled out (Butler & Quarles, 2024; Lareau et al., 2021; Posey-Maddox et al., 2021). Several survey experiments show that the share of Black enrollment does not significantly affect Black parents’ preferences (Hailey, 2022; Mellon & Siegler, 2023), while another suggests that Black parents are particularly willing to both endure longer commutes and give up a same-race majority for a higher ranked school (Ukanwa et al., 2022). The common practice in survey experiments of varying racial composition, however, is insufficient for understanding how Black parents make tradeoffs between documented desires for high-school quality and protecting their children from negative racial climates in their school decisions (Cooper, 2005; Hailey, 2022; Herelle, 2022; Lareau et al., 2021; Posey-Maddox, et al., 2021).

To better understand how Black parents navigate potential tradeoffs between high school quality and denigrating school racial climates, this survey experiment examines how Black parents assess potential schools where Black students have lower academic performance and higher discipline rates than their peers. We randomly assign a large, national sample of Black parents (N = 1,677) to examine a school profile vignette where the school has overall high academic achievement and low suspensions rates but includes either one, both, or neither academic and discipline gaps to assess how test score and suspension disparities affect Black parents’ school preferences and perceptions. Our primary research question then, is to what extent do test score gaps and suspension gaps between Black students and their non-Black peers deter Black parents from choosing schools with higher overall test scores and lower overall suspension rates?

Our results demonstrate that racial disparities in both student discipline and academic outcomes, on average, diminish Black families’ desires to enroll in high-achievement schools and their perceptions of student belonging. These findings align with qualitative research showing that well-resourced, high-achieving schools are less appealing to Black families when they marginalize Black students (Cooper, 2005; Herelle, 2022; Lareau et al., 2021; Posey-Maddox, et al., 2021). Indeed, schools’ unequal punishment of Black students shapes Black parents’ evaluations of potential educational spaces. These findings suggest that, to enhance their appeal to Black families, schools must improve how they teach and treat Black students and close racial gaps in student outcomes. Overall, our findings have implications for understanding Black parent educational decision-making and underscore the limitations of school choice as a tool for racial equity.

## Rational and Positioned Choice: Blending Theoretical Frames

Our survey experiment straddles competing theoretical frameworks for educational decision-making. The rational choice model applies a market logic that frames parents as utility maximizing consumers whose decisions can be modeled as a function of specific school attributes (Eisenlohr et al., 2023). Several survey experiments have employed this frame (Mellon & Siegler, 2023; Ukanwa et al., 2022), which aligns with our fundamental approach of analyzing how parents respond to information. However, our choice of racial outcomes gaps as treatments is grounded in Cooper’s (2005) theoretical framing of positioned choice, which accommodates structural constraints Black parents navigate in their schooling decisions.

The market-based theory of change for school choice policies supposes that competition will improve the quality of public education if parents have more freedom to select options beyond their neighborhood public school (Chubb & Moe, 1990; Friedman & Friedman, 1980; Schneider et al., 1998). School choice advocates argue that parents will choose the highest quality schools as they gather information about schools and choose to satisfy preferences as much as possible subject to constraints such as transportation (Chubb & Moe, 1990). The argument follows that competition should reward higher quality schools and force lower quality schools to close (Hoxby 2003), while also potentially reducing school segregation by decoupling school enrollment from residential segregation (Eisenlohr et al., 2023).

Scholars document that parents’ actual school choices often deviate from rational choice and market-based assumptions. For instance, while choice advocates presume school quality to be schools’ overall academic outcomes or value-added, parents’ school preferences extend beyond academics to include safety, travel convivence, student demographics, and other school factors (Abdulkadiroglu et al. 2020; Billingham & Hunt, 2016; Hailey, 2025a; Harris and Larsen, 2023). Due to time constraints, parents often choose schools based on heuristics or signals of school quality rather full information (Hailey, 2022a; Schneider & Buckley, 2002). Furthermore, scholars caution that choice may fail to increase equitable access to schools or even intensify de facto racial segregation. This can occur to the extent that White parents avoid schools with high shares of students of color, schools try to avoid enrolling students they perceive as more difficult, information gaps persist across racial groups, or racial groups exhibit different preferences or prefer schools that are nearby in segregated neighborhoods (Billingham & Hunt, 2016; Hailey, 2022a; Houston & Henig, 2021; Schneider & Buckley, 2002).

Qualitative studies of Black parents’ school choice processes reveal additional considerations that these parents must weigh. While consuming information and maximizing utility, Black parents navigate ambiguity and choices that are contextualized within the structures and systems surrounding them (Eisenlohr et al., 2023; Herelle, 2022; Lewis-McCoy, 2016; Posey-Maddox, et al., 2021; Waitoller & Super, 2017). Cooper (2005) conceptualized this process as parents as making positioned choices wherein racism, class structures, and sexism are inseparable from a highly emotional, and value-laden school choice process. Herelle (2022, p. 10) quotes a Black mother who, despite being an engineer, said her process for choosing a school “wasn’t the most scientific”. Instead, she decided based on visiting the school and having an intuition that teachers and administrators would care about her Black child. Indeed, scholars document that Black parents, when assessing potential schools, particularly attune to how institutional and interpersonal anti-Black racism may impact Black students’ educational experiences (Lewis-McCoy, 2016; Posey-Maddox et al. 2021).

This study acknowledges both the structural constraints emphasized in positioned choice theory and the focus on information and signaling in rational choice theory by asking if racial disparities serve as an input for Black parents’ educational decision-making. Black parents may be acting rationally by avoiding schools that have disparate student outcomes (even if they may have high test scores). For example, a Black parent may estimate after observing racial disparities in test scores or discipline at a school that the expected value of attending that school is lower for their Black child than for non-Black children (Hailey 2025a; Lewis-McCoy, 2016). Information on lower test scores and higher suspension rates for Black students may signal that the school could marginalize their child both academically and socioemotionally (Lareau et al., 2021). From either the rational or positional perspective, prior survey experiments have overlooked within-school racial inequality as a source of information that may trigger intuitive responses.

## The Importance of Discipline and Academic Disparities in Black Families’ School Evaluations

Black parents pay close attention to both school discipline and academic achievement, when assessing schools. In interviews, Black parents often endorse strict school discipline to the extent that they allow students to focus on academics, promote self-regulation, and create a safe learning environment (Golann et al., 2019; Pattillo, 2015). Simultaneously, however, Black parents also worry that by over-disciplining Black students, schools stigmatize and marginalize Black children (Cooper, 2005; Golann et al., 2019; Herelle, 2022).

Qualitative literature suggests that concerns about excessive discipline may deter some Black parents from choosing schools that would otherwise meet their academic preferences. The explanation for why one Black mother in Butler and Quarles’ (2024) study changed schools typifies the concerns documented in other studies (Cooper, 2005; Herelle, 2022; Lareau et al., 2021). She explained:

What I didn’t like is the way the predominantly White staff yelled at these Black kids. Some of these Black children have parents in prison and you’re rating them, labeling them, based on their behavior … I didn’t like what that was modeling for my son. He had a great education. But I took him out anyway. (Butler & Quarles, 2024, p. 14).

This parent remarked on what they perceived as a racial dynamic and the attachment of problem behavior to students’ identities through labeling. Likewise, she lamented that the punitive approach to behavior management sent a negative message to her Black son about other Black students. Black parents voice strong concerns about schools’ excessive discipline of their own and other Black children, especially as they observe administrators’ resistance to Black parent engagement (Butler & Quarles, 2024; Herelle, 2022; Posey-Maddox, et al., 2021).

Observational and experimental studies substantiate Black parents’ concerns with schools’ disproportionate discipline of Black students. While making up 15 percent of public school enrollment, Black students represent 36 percent of suspensions and expulsions (U.S. Department of Education, 2021). Black students are 3.6 times more likely to be suspended than White students and 3.1 times more likely than Latine students (National Center for Educational Statistics, 2022). Research attributes this discipline disparity to racial bias and stereotyping, with Black students receiving harsher punishments than their peers for the same behaviors (Girvan et al., 2021; Riddle & Sinclair, 2019). For example, Liu et al. (2022) found that when engaged in the same fight, Black students receive harsher punishments than White students. Furthermore, experiments demonstrate that teachers and administrators are more likely to identify Black students as persistent troublemakers and suggest more punitive discipline compared to White students exhibiting similar misbehaviors (Jarvis & Okonofua, 2020; Okonofua & Eberhardt, 2015; Owens, 2022). These harsh disciplinary practices have significant consequences, hindering Black student’s well-being and educational attainment and increasing their risk of criminal legal system involvement (Davison et al., 2021; Morris & Perry, 2016; M. Morris, 2015; Rosenbaum, 2020; Smith et al., 2021; Thompson, 2024).

Black parents also prioritize academic quality when assessing potential schools (Chin, 2022; Hanson et al., 2020; Pattillo, 2015; Schneider et al., 1998; Teske et al., 2006; Ukanwa et al., 2022; Waitoller & Super, 2017). Using enrollment data, Black parents’ top choice schools often have above-average academic outcomes (Abdulkadiroğlu et al., 2020; Chin, 2022; Rowley & McNeill, 2021). Given Black parents’ marginalized economic position, they may particularly emphasize academic performance to promote intergenerational social mobility (Schneider et al., 1998; Ukanwa et al., 2022).

Beyond a general concern for academics in schools, Black parents understand that Black students often have lower academic outcomes than their peers and worry about the role of bias, stereotypes, and discrimination in academic opportunities and teacher expectations (Lewis-McCoy, 2016; Pearman et al., 2019; Posey-Maddox, et al., 2021). Black adults typically attribute Black students doing less well in school than White students to structural factors, such as school resources, than to individual factors such as personal decisions (Alesina et al., 2024; Bañales et al., 2020). This structural concern with Black students’ academic achievement underlies Black parents’ educational decisions. Within predominantly White, high-achieving and suburban schools, Black parents voice concerns about their children being over-identified for special education, under-identified for advanced classes, and overlooked in the classroom (Lewis-McCoy, 2016; Posey-Maddox, et al., 2021). Likewise, they note that teachers often have lower academic expectations for Black students (Butler-Barnes et al., 2019; Rowley & McNeill, 2021). Parents also link schools’ hyper-discipline of Black students with lower academic achievement and racial achievement gaps (Posey-Maddox et al., 2021)—a connection supported by a recent study of US schools (Pearman et al., 2019). Black families often seek schools where Black students specifically achieve and thrive academically (Rowley & McNeill, 2021) and view racial disparities in outcomes as a signal for schools’ racialized structures (Lareau et al., 2021; Lewis-McCoy, 2016).

Scholars have proposed that Black parents’ awareness of anti-Black racism in schools prompts them to engage in *racialized risk assessments* of potential schools (Posey-Maddox et al., 2021). These assessments involve evaluations of schools’ “racial disparities in teacher expectations and academic outcomes…systems of discipline in the schools … how teachers and school personnel made decisions based on negative stereotypes” (p. 49; Posey-Maddox et al. 2021).

## The Current Study

Guided by qualitative documentation of Black parents’ concerns with how schools discipline and teach Black students, in this study, we examine whether racial disparities in test scores and suspensions deter Black parents from higher-achievement schools. Our survey experiment allows us to manipulate school suspension gaps and test score gaps independent of school racial composition, to better understand how Black parents perceive schools with high overall academic outcomes but where Black students have lower academic achievement and higher disciplinary rates than their peers.

This study contributes to our larger understanding of how educational racial inequities shape Black families’ perceptions of schools. Asking Black parents about their preferences directly can reduce ambiguity about what they value in schools and guard against deficit perspectives that perhaps they do not value academic achievement enough to send their children to ostensibly “better” schools farther away from home. In addition to structural barriers to accessing these schools (Eisenlohr et al., 2023), Black parents may respond to considerations about school climate that are not race-neutral but depend on their positionalities and understanding of how schools often marginalize Black students (Cooper, 2005).

## Method

### Experimental Design

We recruited Black parents in the U.S. from the Centiment survey panel. Centiment maintains a panel by recruiting people from social media and creating panelist profiles. Centiment only invited panelists to our survey who self-identified as Black parents.

We pre-registered our experiment on the Open Science Framework (https://osf.io/4zegs) and collected two waves of data: one in June 2023 (N = 776 Black parents) and one in February 2024 (N = 901 Black parents). Respondents in both waves encountered the same vignette:

Please imagine that your oldest child is about to enter middle school for the first time this fall. You have two public middle schools to choose from: Walker and Prim.

You visit your state’s official school directory and see the school profiles for Walker and Prim on the next page. Please consider the two school profiles. Then you will answer questions about your preferences.

The next page showed respondents two school profiles. One profile—Walker Middle School—is an anchor with all details held constant regardless of random assignment (Appendix Fig. 4). This anchor allows all respondents to picture an alternative to Prim, the focal school. It is not a control group or an arm of the experiment because it does not vary across respondents.

**Fig. 1:**
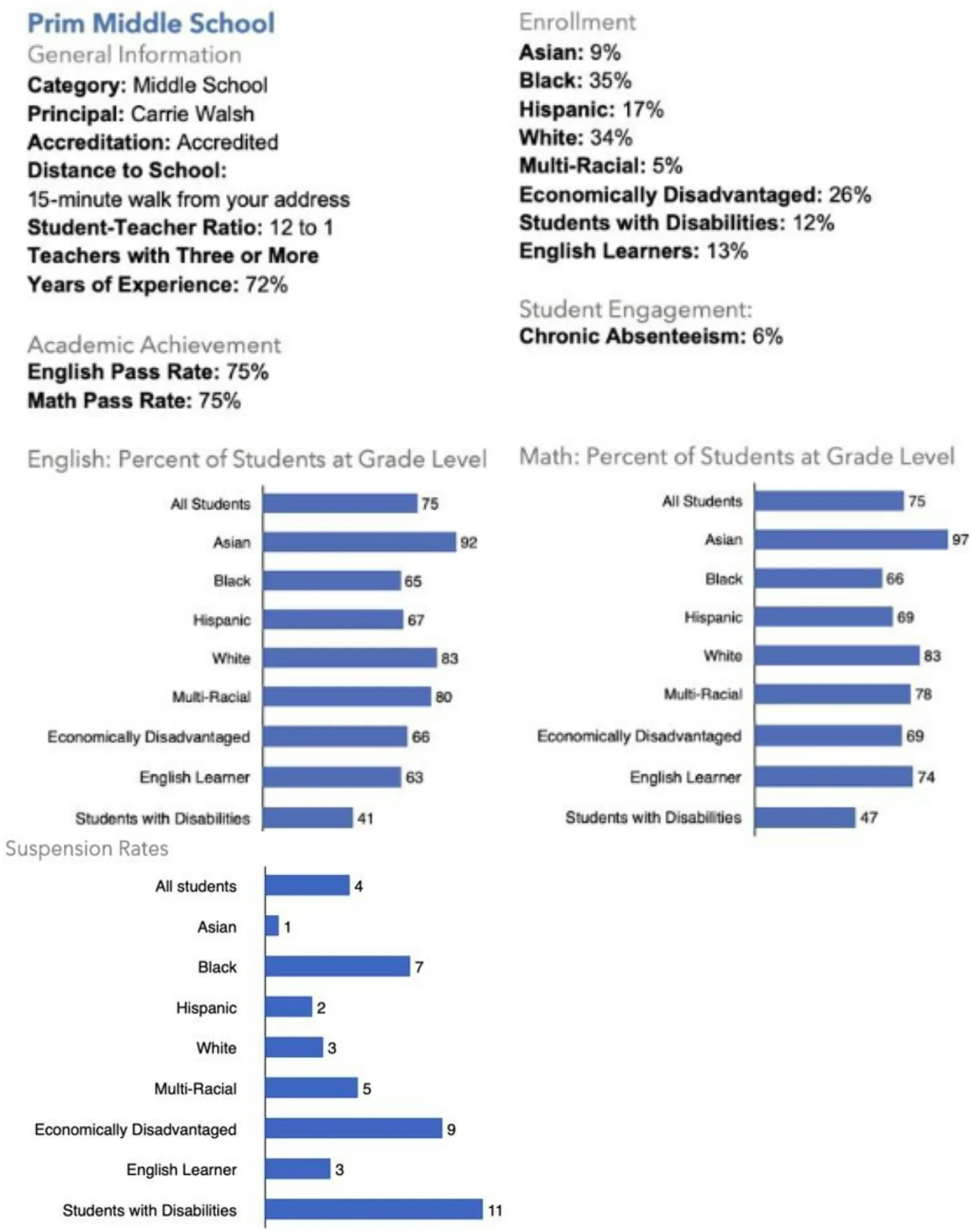
Example Prim Middle School Profile

The other profile was for the focal school—Prim Middle School—whose profile was randomly assigned to a control or one of three treatment conditions shown in Table 1 below. Notably, in all conditions, Prim has smaller proportions of students who are Black, Hispanic, lower-income, and English-learners than Walker. See Appendix Table 5.^1^ The profile for Prim when a participant is assigned to both gaps condition is shown in Fig. 1 below.

**Table 1:** Randomly Assigned Conditions for Gaps between Black and non-Black students at Prim Middle School

No Gap (Control)	Test Score Gap Only (Treatment 1)
Suspension Gap Only (Treatment 2)	Test Score and Suspension Gap (Treatment 3)

Regardless of the randomly assigned condition, Prim Middle School is an overall higher quality school than Walker. Prim has overall higher test score proficiency and lower overall suspension rates. Teachers at Prim also have more years of experience and students have lower absenteeism rates.

In the control condition, Prim has no gap between Black students’ average and the overall school average across all racial/ethnic groups in either test scores or suspension rates. In the treatment conditions, the Prim profile has either (1) a test score gap only—Black students have lower math and reading test score proficiency rates than their peers, (2) suspension gap only—Black students have higher suspension rates than their peers, or (3) gaps in both test scores and suspension rates.^2^ Appendix Table 5 includes the consistent school profile information used for all Walker and Prim Middle Schools in Panel A and the Prim School information that varied by treatment status in Panel B.

Black students have lower test scores and higher suspension rates than their White peers in most districts (Pearman et al., 2019). Although the treatment arm with both racial outcomes gaps represents the status quo, this study offers practical value in including separate treatment arms with only suspension gaps and only test score gaps. We hypothesize that the combined effect of test score gaps and suspension gaps may deter Black parents more than the two separate effects. Researchers have hypothesized that anti-Black biases among teachers and low feelings of belonging among Black students may explain why higher suspension rates were especially predictive of lower test scores for Black students (Chin et al., 2020; Pearman et al., 2019). Analogously, our study asks whether Black parents form more negative reactions when schools have both test score and suspension gaps. Understanding the independent and additive effects of academic and discipline disparities on Black parents’ school perceptions and preferences is especially important, as practitioners and policymakers may need multifaceted, equity-focused strategies to address both test score and suspension gaps (see Fryer & Howard-Noveck, 2020; Kraft, 2015; Welsh, 2023; Welsh & Little, 2018).

After viewing the profiles, respondents were asked about their desire to enroll in Prim Middle School and their perceptions of anticipated belonging at the school. The items are described in the Measures section below and described in detail in Appendix A. The choice question was displayed on one page, and parents could not go back to change their answers after moving on. We did this so that the questions about expected student belonging that appeared on the next page could not prime or otherwise influence parents’ hypothetical school choice.

The experiment was the same in the two survey waves with three notable exceptions^3^. First, the initial wave asked parents whether they would like to view school suspension rates. We included this step because test scores often appear front and center on state and district school search websites, whereas discipline rates can require additional clicks. The second wave removed this step and simply showed parents the school suspension rates. We made this change to eliminate the possibility that asking parents if they wanted to see suspensions might prime them to focus more on the suspension rates than they otherwise would. Together, wave one sheds light on a context when parents have to look for suspension data by race while wave two represents contexts where suspension data by race are readily presented. Second, the follow up wave added a series of questions about parent expectations of student belonging. Third, the last question in the second wave asked parents to indicate which factors from a predefined list that influenced their answers to previous questions about the schools. They could mark all that apply for eight different factors: math test scores, reading test scores, suspension rates, gaps in student outcomes, distance, teacher experience, absenteeism, and diversity. Parents did not have the option to go back and change prior answers because we did not want to prime them by listing “gaps in student outcomes” as an option.^4^ For items collected in both waves, we pool the sample across waves to maximize statistical power, but we report regression analyses separately by wave in Appendix Tables 9 and 10.

### Sample

Table 2 describes the full sample pooled across waves. Most of the parents identified as Black only (91.6 percent), though some were multiracial. About two-thirds of respondents were between the ages of 25 and 44, one-third were married, and a quarter completed a BA degree or higher. Nearly half of respondents resided in urban areas and more than a third lived in the South. Randomization was fairly successful in achieving balanced demographics across treatment groups; see Appendix Table 6.

**Table 2:** Sample Descriptives (Pooled Across Waves)

Race/ethnicity	
Black only	91.6%
Black multiracial/ethnic	8.4%
Male	50.1%
Age	
Under 25	8.8%
25–34	31.9%
35–44	36.6%
45–54	16.5%
55+	6.2%
Marital/cohabitation Status	
Married	33.2%
Cohabiting	20.3%
Number of children in home	1.88
Highest level of education	
BA degree or higher	22.7%
Associates degree	11.1%
Some college	25.9%
High school	29.9%
Less than High school	5.5%
K-12 teaching experience	
Previously taught K-12	10.4%
Teaches K-12 now	3.8%
Locale	
Rural	27.7%
Suburban	25.0%
Town	0.3%
Urban	47.0%
Region	
Northeast	20.4%
Midwest	25.4%
South	38.2%
West	15.0%
N	1677

Not all categories sum to 100 because some category levels are not shown. For example, divorced is not shown for marital status and trade school is not shown for education

While our sample derives from an online sample, it somewhat represents Black parents nationwide. Our respondents resembled the population of US Black adults in marital status and urbanity but had slightly lower proportions of multiracial people, BA completers, and Southern US residents. Notably, researchers have found comparable effects from information treatments across paired survey experiments using online samples and nationally representative samples (Berinsky et al., 2012; Thomas & Clifford, 2017). See Appendix A for additional methodological details on covariate balance and sample comparison to the population of US Black parents.

### Measures

#### Desire to Enroll

Our pre-registered, confirmatory outcome is a factor score based on a six-item scale for a construct we named desire to enroll. This score captures parents’ desire to enroll their child in Prim Middle School, the focal school we experimentally manipulated. The items that comprise the scale are listed in Appendix Table 8 Panel A along with their factor loadings. We developed the items iteratively from the school choice literature and piloting. In our final scale, two items represent a discrete choice (whether to enroll in Prim, whether to choose Prim over Walker), three capture emotions (excitement, enthusiasm about Prim’s fit, and disappointment), and one considers desire to avoid Prim. See Appendix for more details.

Alongside our confirmatory factor score outcome, we also report results for the two items that represent discrete choices to ease interpretation. We dichotomized both into binary measures for whether the parent would enroll in Prim and whether the parent would choose Prim over the anchor school (“Very Likely”/“Somewhat Likely” vs. “Very Unlikely”/“Somewhat Unlikely”).

#### Expected Belonging

As an exploratory outcome in Wave two, we examined effects of racial disparities on a factor score for parents’ expectations of student belonging (see Hailey and Murray, 2025). The expected belonging scale consists of three items. The first assessed how welcome respondents believed their child would feel at Prim using a 4-point Likert scale from “Very Welcome” to “Very Unwelcome.” The other two ask how likely their child would be to make friends and be socially and emotionally supported at Prim on a 4-point scale from “Very Unlikely” to “Very Likely.” Appendix Table 8 Panel B provides the full items and their factor loadings.

### Estimation

To answer our research question—how do test score gaps and suspension gaps between Black students and their peers affect Black parents' likelihood of choosing a prospective school—we estimate the influence of test score gaps, suspension gaps, and both gaps on Black parents’ school preferences and expected belonging using a OLS regression with wave-fixed effects (Eq. 1). This regression estimates the differences in means between the control group and each treatment group, controlling for respondent background characteristics. For Wave 1, the differences in means represent intent-to-treat (ITT) effects since most but not all (~ 75%) of parents clicked to view suspension rates. For Wave 2, there is no non-compliance since all parents were shown the suspension rates.

$$\:\left(1\right)\:{Y}_{it}=\:{\beta\:}_{0}+\:{\beta\:}_{1}{Test\:Score\:Gap}_{it}+\:{\beta\:}_{2}{Suspension\:Gap}_{it}+\:{\beta\:}_{3}{Suspension\:Gap\:and\:Test\:Score\:Gap}_{it}+{X}_{i}+\:{\gamma\:}_{t}+\:{\epsilon\:}_{it}$$

where, $\:{Y}_{it}$ is a factor score for desire to enroll in Prim Middle School (or alternatively the likelihood of enrolling or choosing Prim and expected belonging at Prim) for parent *i* in data collection wave *t*, *Test Score Gap* indicates random assignment to a school profile with the test score gap, *Suspension Gap* indicates random assignment to a profile with the suspension gap, and $\:Suspension\:Gap\:and\:Test\:Score\:Gap$ indicates random assignment to a profile with both test score and suspension gaps. *X* represents a vector of covariates included to increase precision and account for slight differences in respondent characteristics across treatment groups: an indicator for multi-racial Black, age, gender, education, marital/cohabitation status, currently or previously being a K-12 teacher, and locale (rural, town, suburban, city) and US region based on zip code designations by the US Census Bureau (Geverdt, 2019). The regressions include fixed effects $\:{\gamma\:}_{t}$ at time *t* for each wave of data collection.

## Results

### Effects of Suspension and Test Score Gaps on School Preferences and Perceptions

**Fig. 2:**
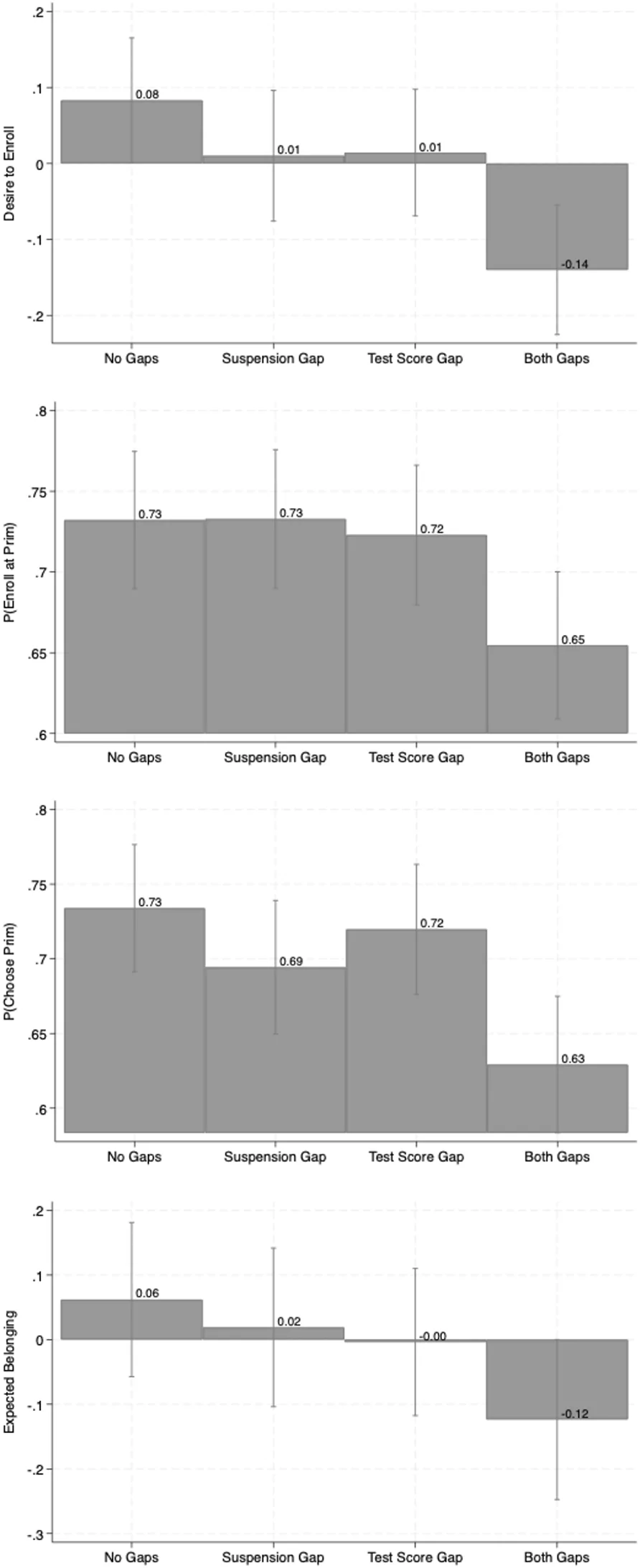
Adjusted Means and 95 percent confidence intervals of Outcomes by Treatment Group. Average adjusted means and 95 percent confidence intervals from OLS models in Columns 1,2, 3, and 6 of Table 3

Suspension and test score gaps deter Black parents’ desires to enroll their students in schools with overall higher-academic achievement levels. Table 3 shows dummy coded regression results comparing each treatment to the control in the pooled sample. These results can be interpreted as the difference in means between each treatment condition relative to the control condition, accounting for respondent background.^5^ Fig. 2 shows the adjusted means and 95 percent confidence intervals for each outcome by the control and treatment groups, as derived from the regressions in Table 3.

**Table 3:** Effects of Suspension and Test Score Gaps on Desire to Enroll and Choice

	Pooled Sample			Wave 1	Wave 2	
	(1)	(2)	(3)	(4)	(5)	(6)
Variables	Desire to Enroll	P(Enroll at Prim)	P(Choose Prim)	Desire to Enroll	Desire to Enroll	Expected Belonging
No Gap (reference)						
Suspension Gap	−0.073	0.001	−0.034	−0.152*	0.000	−0.043
	(0.061)	(0.031)	(0.032)	(0.091)	(0.082)	(0.087)
Test Score Gap	−0.069	−0.009	−0.014	−0.047	−0.084	−0.066
	(0.060)	(0.031)	(0.031)	(0.088)	(0.083)	(0.084)
Suspension and Test Score Gap	−0.223***	−0.078**	−0.105***	−0.250***	−0.217***	−0.186**
	(0.060)	(0.032)	(0.032)	(0.089)	(0.084)	(0.088)
Constant	0.015	0.790***	0.736***	0.155	−0.090	−0.007
	(0.145)	(0.074)	(0.075)	(0.269)	(0.171)	(0.180)
Observations	1,675	1,675	1,674	774	901	901
R-squared	0.033	0.023	0.028	0.044	0.050	0.036

Robust standard errors in parentheses *** p<0.01, ** p<0.05, * p<0.1

Columns 1-3 show results from OLS regressions estimating Equation 1 in both waves of data collection with a fixed effect for wave. Column 1 shows effects on our factor score for desire to enroll, which is approximately normal. Columns 2 and 3 show effects from linear probability models on the likelihood of enrolling at Prim or choosing Prim over the alternative anchor school. Column 4 shows results from wave 1 and columns 5-6 shows results from wave 2. The covariates are an indicator for whether the parent was male, an indicator for identifying as multi-racial in addition to Black, an indicator for having a BA degree or higher, an indicator for being married, and indicator for cohabiting, an indicator for currently or previously being a K-12 teacher, indicators for locale (suburban, town, rural, urban), and age group.

Column 1 in Fig. 2 and Table 3 shows that the suspension and test score gap treatment significantly reduced Black parents’ desire to enroll in Prim. Parents in the “No Gaps” control group rated Prim at.08 standard deviations on the desire to enroll factor, while parents in the “Suspension and Test Score Gap” treatment rated Prim at −.14 standard deviations. The coefficient for the suspension and test score gaps in Table 3 (*b*=-.22) means that, on average, the presence of both gaps reduced parents desires to enroll in Prim by.22 standard deviations compared to parents who reviewed schools without either gaps. Parent’s desires to enroll in Prim did not differ significantly between the test score gap only condition and the control condition nor between the suspension gap only condition and the control condition (see column 1 in Table 3, *b*^*test score−gap*^= −0.07,*b*^*suspension−gap*^= −0.07,p >.23).

Columns 2 and 3 of Fig. 2 and Table 3 include regression results for two binary outcomes: the likelihood of enrolling and the likelihood of choosing Prim over the alternative anchor school. These outcomes followed similar patterns as the desire to enroll factor. Column 2 in Fig. 2 demonstrates that about 73 percent of the parents in the “No Gaps” control condition reported that they would be “Somewhat likely” or “Very likely” to enroll their child at Prim Middle School. The means for the test score gap treatment and suspension gap treatment are similar to the control group. Table 3 also shows that the cofficients for theses treatments are small and not statistically significant, suggesting that test score gaps and suspension gaps in solitaire may not substantively shift Black parents’ likelihood of enrolling in overall high-achieving schools. However, parents who observed schools with both suspension and test-score gaps were 8 percentage points less likely to say they would enroll at Prim than those in the control group (see column 2 of Fig. 2 and Table 3, *b*=−0.077,p <.05). Column 3 of Fig. 2 also demonstrates that Black parents had significantly lower likelihood of choosing Prim when Black students had *both* lower academic performance and higher exclusionary discipline rates than their peers. Relative to the control group, Black parents who observed schools with both test score and suspension gaps were 10.5 percentage points less likely to choose Prim (see column 3 of Table 3, *b*=−0.105,p <.01).

The subsequent columns of Table 3 look at results separately by wave of data collection. We found that in Wave one (column 4), when families had to opt into viewing suspension rates, the suspension gaps in solitaire and the combined suspension and test score gaps significantly reduced parents’ desires to enroll (*b-suspension gap*=−0.152,p <.1; *b-suspension and test score gap*=−0.250). Expanded results in Appendix Table 9 show that, in Wave one, suspension gaps treatment and the both gaps treatment reduced the likelihood of choosing Prim over the alternative by 9 and 14 percentage points, respectively (*b-suspension gap*=−0.0897,p <.1;*b-suspension and test score gap*=−0.139,p <.01).

In Wave two, when suspension rates were showed up front, only the combined suspension and test score disparities had a large and significant effect on parents’ desires to enroll and expectations of belonging (see column 5 and 6 in Table 3). The effect of suspension and test score gaps lowered parents’ desires to enroll in Prim by.22 standard deviations (*b-suspension gap and test score gap*=−0.217,p <.01). Furthermore, Black parents anticipated that their child would experience less belonging in schools where Black students had lower test scores and higher suspension rates than peers. Compared to the “no gaps” control group, Black parents in the test score and suspension gaps treatment rated Prim.19 standard deviations lower on expected belonging (see column 6 of Table 3, *b*=−0.186,p <.05).

While it is possible that asking parents whether they would like to view suspensions primed them and led to larger effects of suspension gaps in Wave one, the consistent negative direction of the test score and suspension gaps coefficients in Wave two suggests that priming may not fully underlie the effect of suspension disparities in Black parents’ school assessments. Instead, it is likely that both test score and suspension gaps have negative effects that vary by context.

Overall, we find that Black families are less likely to want to enroll in a school with higher overall academic outcomes when the Black students in the school have both lower academic outcomes and higher suspension rates than their peers. When both disparities are present, they also believe that their Black student would experience less socioemotional support and welcomeness in the higher-achievement school.^6^

### Motivations for Parents’ Tradeoffs

**Fig. 3:**
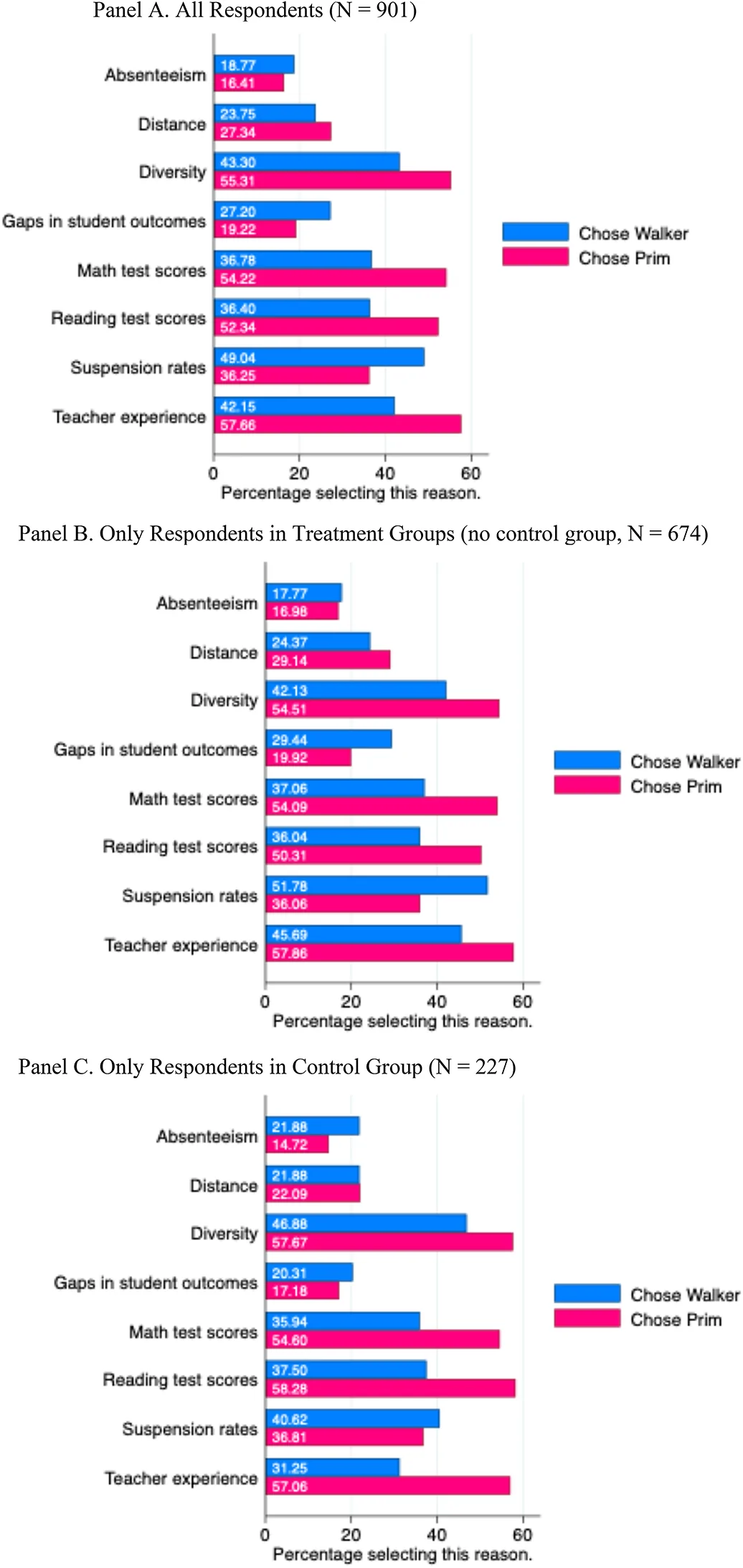
Reasons for Respondent Answers in Wave 2 by Which School They Preferred

To better understand Black parents’ tradeoffs when considering a school with overall higher academic outcomes but where Black students have relatively lower academic achievement and higher suspension rates than their peers, we assess descriptive results asking respondents to delineate what factors influenced their school preferences. We added this question in the second wave of data collection. These results are shown in Fig. 3, separately depending on which school parents would be more likely to choose.^7^

Panel A shows that, compared to parents who chose Walker, parents who chose Prim were generally more likely to mark that teacher experience, math and reading test scores, and diversity motivated their school preferences (differences in means significant at p <.001). These results align with the experiment design since Prim had overall higher teacher experience and test scores and larger Asian, White, and multi-racial student populations than Walker, no matter the experimental condition (see Appendix Table 5).

Notably, parents who choose Walker over Prim despite Prim’s overall higher academic achievement particularly noted that *suspensions* and *gaps in student outcomes* motivated their preferences. Panel A demonstrates that nearly half of the parents who choose Walker indicated that suspensions played a role in how they perceived the schools—a significant higher proportion than parents who choose Prim (36 percent; difference significant at p <.001). Moreover, parents who selected Walker were more likely to emphasize that gaps in student outcomes motivated their decisions (27 percent for Walker-choosers compared to 19 percent for Prim-choosers; difference significant at p <.01). These patterns were especially salient in the treatment conditions where Prim had suspension gaps and/or test score gaps; see Fig. 3, Panel B. This descriptive analysis suggests that Black parents who choose to trade-off a school with overall higher test scores for a school with overall lower academic outcomes may be particularly attuned to schools’ discipline culture and Black students’ experiences relative to their peers.

## Discussion, Limitations, and Implications

Parents weigh multiple factors when considering potential schools (Golann et al., 2019; Posey-Maddox et al., 2021; Saporito & Lareau, 1999). Given persistent racial disparities in Black student achievement and exclusionary discipline (Pearman, 2019), qualitative studies suggest that Black parents adopt a hypervigilant approach when evaluating schools, closely examining Black students' potentially marginalized experiences relative to their peers (Butler & Quarles, 2024; Clerge, 2022; Lewis-McCoy, 2014; Pattillo, 2015; Posey-Maddox et al., 2021). By employing a novel school choice survey experiment among a national sample of Black parents, we are able to isolate whether racial disparities have a casual effect on Black parents’ school preferences and perceptions, in a way not possible with interview-based and observational data. We uniquely demonstrate that in overall high-achievement schools, the hyper-discipline and lower academic achievement of Black students relative to their peers significantly reduce Black parents’ preference for schools and beliefs that their student would feel supported and welcome. Test score and suspension gaps reduced parents’ desire to enroll in high-achievement schools by 0.22 standard deviations. Moreover, these racial disparities diminished parents’ likelihood of choosing the high-achievement school by 10.5 percentage points.

While our findings suggest that racial disparities independently shape Black parents’ school preferences, we acknowledge several key limitations of this experimental design. Our experiment is low stakes, which may lead to inattention to the treatment. Although all respondents in our analysis passed an attention check question, analyses based on response-time attentiveness clustering showed variation in potential attentiveness (Read et al., 2021, 2022; see Appendix Table 12). Importantly, highly inattentive respondents might attenuate our study’s treatment effects if they did not notice the disparities that represent the treatment conditions. Indeed, according to response-time clusters, effects appear concentrated among respondents with potentially higher attentiveness (see Appendix Fig. 6). As such, our results likely reflect lower-bound estimates of the effects of test score and suspension disparities on Black parents’ school preferences.

Comparing school profiles, as parents did in this study, likely represents only one aspect of families’ complex school evaluation processes (Bell, 2009; Lareau et al., 2021). Although our experiment captures one strategy in Black families’ racialized risk assessments—examining school-level profiles with student outcomes by race—their concerns with school racial inequalities likely arise through other approaches (Lareau et al., 2021). Black parents may gather knowledge about school racial inequalities through school visits or word of mouth (Lewis-McCoy, 2016). Moreover, concerns with school racial climate likely interact with parents’ understanding of their child, perceptions of schools’ broader reputations, and the experiences of Black people in their local community (Posey-Maddox et al., 2021; Rhodes et al., 2023). This study does not assess how school racial disparities compare in importance to other characteristics in Black parents’ preferences. Future research could use conjoint experiments to examine how Black families weigh multiple dimensions of school quality, when making school choices. Future studies should also further explore how Black parents incorporate information about schools’ racial disparities into their actual school evaluations and selections. This could include qualitative studies with high and low-income Black families choosing schools (see Lareau et al., 2021) and quantitative analyses of Black families’ actual school applications that account for overall school quality and Black-student-specific outcomes (see Hailey, 2025b).

Our confirmatory findings—that racial disparities reduce Black parents’ desire for otherwise higher achievement schools—advances our understanding of racialized educational decision making. Theoretically, this study offers further evidence that, when making school choices, parents consider school quality indicators beyond overall test scores and value-added (Abdulkadiroglu et al. 2020; Beuermann et al. 2023). Specifically, it emphasizes that the hyper-discipline and lower-achievement of Black students shape how Black families evaluate educational institutions (Cooper, 2005; Lareau et al., 2021; Lewis-McCoy, 2014; Posey-Maddox et al., 2021). These results point to an alternative definition of high-quality schools—one centered on whether schools marginalize or support Black students.

Black parents’ educational choices should be understood as bound by a racialized educational structure. In the face of discriminatory and unequal school environments, Black parents may be rational utility maximizers— avoiding schools with signs that their child may not be well-served academically or socioemotionally (Posey-Maddox et al., 2021). What might appear to be suboptimal decisions—such as bypassing high-achieving schools—can instead reflect Black parents’ strategic navigation of institutional inequality and anti-Black racism (Lareau et al., 2021; Lewis-McCoy, 2014). These choices only seem suboptimal when Black students’ distinct experiences are ignored. This suggests that evaluations of parents’ school choices—and the assumptions underlying school choice policies— may be misleading if they fail to account for structural inequality and students’ positionality in the social structure (Cooper, 2005; Lareau et al., 2021).

By engaging Black voices in this study, we glean a deeper understanding of the limits of school choice as a tool for racial equity (Cooper, 2005). The prevalence of racial discipline disparities and achievement gaps (Pearman et al., 2019)—better yet—educational debts (Ladson-Billings, 2006) might explain why, when given the choice, some Black parents are hesitant to enroll in highly-resourced, higher-achieving schools (Eisenlohr et al., 2023; Lewis-McCoy, 2016). While addressing structural barriers that hinder Black families access to high-quality schools (i.e. transportation and residential segregation), policymakers seeking to advance educational equity through school choice landscapes must also address within-school racial inequality and practices that marginalize Black students.

Prior studies suggest several strategies that districts could employ to reduce Black-White achievement and discipline disparities. Interventions such as high-dosage tutoring, class-size reduction, and hiring HBCU-trained teachers have boosted test scores for Black students, being particularly effective for those with lower initial test scores (Edmonds, 2022; Fryer & Howard-Noveck, 2020; Kraft, 2015; Krueger & Whitmore, 2001; Shin, 2012). While recent policy efforts have lowered the overall use of school suspensions, few interventions have diminished Black-White suspension disparities, as efforts to reduce suspensions often disproportionately benefitted White students (Welsh, 2023; Welsh & Little, 2018). To mitigate persistent racial inequities in school discipline, policymakers must implement reforms that explicitly address the anti-Black biases and stereotypes driving the hyper-discipline of Black students (Jarvis & Okonofua, 2020; Okonofua & Eberhardt, 2015; Owens, 2022; Welsh, 2023). Additionally, hiring more Black teachers could improve both academic and disciplinary outcomes for Black students. Research shows that Black teachers are less likely to suspend Black students (Holt & Gershenson, 2019; Lindsay & Hart, 2017) and positively impact their short and long-term academic achievement (Gershenson et al., 2022). Systematic reforms to reduce racial disparities in academic performance and discipline will not only enhance Black students’ socioemotional well-being and long-term outcomes but, as this study suggests, also increase the number of schools perceived as viable options for Black families.

## Supplementary Information

Below is the link to the electronic supplementary material.

## Footnotes

1 It is important to note that, in Walker, there is no gap between Black students’ test-scores and suspension rates and the academic achievement and exclusionary discipline rates for all students.

2 Recent work suggests that displaying test score growth instead of status may prompt White parents to consider more racially diverse schools, we used test score proficiency because it remains ubiquitous on school performance profiles whereas growth data are supplemental (Houston & Henig, 2023).

3 The second wave also corrected a small typo (a floating number “15”) in the school profile with the test-score gap treatment (See Appendix Fig. 5). The typo did not cover any information being experimentally manipulated, nor did parents in cognitive interviews mention it.

4 Based on the treatment manipulations, we would expect that parents who received the test score and/or suspension gaps treatments would be more likely to indicate that "Gaps in student outcomes" motivated their choices than those in the “no gaps” control group. Aligning with that expectation, parents who viewed school profiles with test score and/or suspension gaps were 5 percentage points more likely to indicate that gaps in student outcomes motivated their choices (p <.10).

5 Results are substantively similar using dummy-coded regressions that do not control for covariates.

6 These significant differences between the both-gaps treatment and the no gaps control reflect the combined effect of the suspension gap, the test score gap, and any interaction effect of the two gaps. See Appendix for results from effect-coded OLS regressions that parse out these effects. Effect coded regressions suggest that disciplinary and test score gaps contributed equally to Black parents’ lower desires to enroll in the school with both suspension and test score gaps relative to the school with no gaps.

7 This delineation is based on the question “I would choose Prim Middle School over Walker Middle School.” We coded respondents who answered *very unlikely* or *somewhat unlikely* as choosing Walker and those who answered *somewhat likely* or *very likely* as choosing Prim. Overall, 31 percent of parents chose Walker and 69 percent chose Prim; see Fig. 2, column 3 for proportion who chose Prim by the treatment condition.
