# Supplementary material for: Racial Disparities and Black Parents’ School Preferences: Evidence from a Survey Experiment
Source: Urban Rev. 2026 Jan 3;58(1):11. doi: 10.1007/s11256-025-00797-x (PMC12764616; doi:10.1007/s11256-025-00797-x)
Supplement: Supplementary file 1 — Supplementary material 1 (DOCX 576.4 kb) [file 11256_2025_797_MOESM1_ESM.docx]

**Appendix A. Additional Methodological Details**

**Covariate Balance.** Overall, randomization was fairly successful in producing covariate balance. The balance table in Table 6 shows results from regressing the effect-coded treatment variables on baseline covariates in the pooled sample. The F-test statistics and p-values for joint significance assess whether the treatment assignment predicts each covariate. In the pooled sample across waves, two of the 25 F-statistics (8%) were statistically significant at the 0.10 level. This is slightly more than the 5% expected by chance and is driven by differences across experimental conditions in the distribution of age and locale, specifically whether the respondent lived in a suburb. To account for the slight imbalances in the pooled sample, we include covariates in our regressions, consistent with our pre-registration plan.

**Survey Sample Comparison to National Population of Black Parents.** Table 7 compares demographics for the sample pooled across waves to population estimates for the most similar categories available from the U.S. Census Bureau’s American Community Survey (ACS) microdata for 2022 and the National Center for Education Statistic’s (NCES) Public Elementary/Secondary School Universe Survey for 2019-2020 (Moslimani et al., 2024; National Center for Education Statistics, 2021a, 2023). Our sample generally has similar characteristics of Black parents nationwide, with a few exceptions. Compared to Black adults nationwide, the survey sample had a slightly lower share of multiracial individuals, lower shares of bachelor degree completers, fewer suburban and town residents, and more rural residents. Finally, a third of our sample resided in the South compared to more than half the adult Black population.

Notably, researchers have found comparable effects from information treatments across paired survey experiments using online samples and nationally representative samples (Berinsky et al., 2012; Thomas & Clifford, 2017). Prior research suggests that data quality from online panels is comparable to that of Mturk (Kennedy et al., 2020; Peer et al., 2022; Smith et al., 2016), which has been used for survey experiments in education (Houston & Henig, 2021). We decided on Centiment, however, because having a pre-screened panel helped ensure that respondents were Black.

**Details on Desire to Enroll Scale.** From the school choice literature, we developed a construct map for desire to enroll with multiple levels reflecting both choices and emotions. For example, middle-class Black parents expressed apprehension about avoiding low-performing schools (Lareau et al., 2021). Some Black and White middle-class parents second-guessed their choice of an urban public school (Cucchiara, 2013). Low-income parents in a system of ranked school choice felt frustrated and disappointed when they registered late—often amid housing instability—and got assigned to leftover schools that they had not ranked (Fong & Faude, 2018). On the one hand, some parents did not actively consider or compare schools when choice within the district was available but enrolled in their zoned school by default (Goldring & Hausman, 1999). On the other hand, a minority of parents with the financial means to enact their desires chose where to live based on the public schools (Cuddy et al., 2020; Kimelberg, 2014; National Center for Education Statistics, 2021b). We did not want our survey items to conflate desire with financial means, as in prior survey experiments that told parents they would have to pay tuition or move to avoid the hypothetical school (Billingham et al., 2020; Billingham & Hunt, 2016). We also concluded that whether one item about whether parents would enroll in a school might not distinguish those with a strong desire to attend or avoid the school from those who found it acceptable.

We piloted five items intended to allow range in desire to enroll—from aversion to acceptance to enthusiasm—with a convenience sample of 17 graduate students at a Southeastern University and in cognitive interviews with three Black parents. With the pilot feedback and item-total correlations, we retained “Avoid”, “Disappoint,” and “Enroll”; changed an item about ranking schools into an easier-to-understand item about choosing between schools; and added two items.

We computed desire to enroll factor scores separately for each wave based on item analyses and our preregistration; see Appendix Table 8 for the factor loadings. Note that we reverse coded the negatively worded items (Disappoint and Avoid) so that lower numbers (e.g., 1 “Strongly Disagree”) represented a high desire to enroll, consistent with the other items. The eigenvalues suggested a two-factor solution, where Excited, Fit, Choose, and Enroll loaded more onto one factor while the negatively worded items were more correlated with each other. Still, the one-factor solution explained most of the variation. It had moderate factor loadings for the negatively worded items, high factor loadings for the other items, and strong internal consistency (Cronbach’s alpha = 0.81 in Wave 1 and 0.82 in Wave 2). Thus, we chose a single factor that was weighted more toward the positively worded items but used all six items, as registered in our pre-analysis plan.

**More details on Effect Coding.** We estimate the influence of test score gaps and suspension gaps on Black parents’ school preferences using OLS regression and effect coding (Equation 2), which maximizes statistical power for estimating main effects and interaction effects in a factorial experiment by splitting the sample roughly in half for each comparison (Kugler et al., 2018).

$$\left( 2 \right) Y_{i}= \beta_{0}+ \beta_{1}Test Score Gap+ \beta_{2}Suspension Gap+ \beta_{3}\left( Test Score x Suspension Gap \right)+X_{i}+ \varepsilon_{i}$$

where, $Y_{i}$ is a factor score for desire to enroll in Prim Middle School (or alternatively the likelihood of enrolling or choosing Prim), *Test Score Gap* indicates random assignment to a school profile with a test score gap, *Suspension Gap* indicates random assignment to a profile with a suspension gap, and $Suspension Gap x Test Score Gap$ is an interaction term indicating that both gaps were presented. *X* represents a vector of covariates included to increase precision and account for slight differences in respondent characteristics across treatment groups: age, gender, education, marital/cohabitation status, and locale (rural, town, suburban, city) based on zip code designations by the US Census Bureau (Geverdt, 2019).

Effect coding represents the no gap condition as [-1,-1], the suspension gap only treatment as [1, -1], the test score gap only treatment as [-1, 1], and the both gaps treatment as [1,1]. Effect coding dictates the interpretation of the estimates from Equation 2. The intercept $\beta_{0}$ represents the grand mean, or the unweighted average of the outcome across conditions in the sample. Since the treatments in this study have two-levels, -1 and 1, the effect-coded regression coefficients represent half the change in the dependent variable when switching between the levels or, equivalently, the deviation from the grand mean (Brehm & Alday, 2022). The coefficient $\beta_{1}$ represents half the effect of a test score gap, regardless of whether there was a suspension gap. Likewise, $\beta_{2}$ represents half the effect of a suspension gap, averaged across parents in our sample with and without test score gaps. Finally, $\beta_{3}$ represents one fourth of the interaction effect. In order to interpret both the main and interaction effects as treatment effects instead of deviations from the grand mean, we multiply the coefficients by a scaling constant of 2; this does not affect hypothesis testing because it applies to the standard errors as well (Kugler et al., 2018).

Table A11 reports the results of the effect coding regressions and shows the main and interaction effects of suspension gaps and test score gaps on the desire to enroll factor score, the likelihood of enrolling, and the likelihood of choosing Prim over the alternative anchor school. The two columns on the far right remind readers that the effect coding computes each coefficient by comparing the mean of the two pooled experimental conditions that are shaded to the mean of the two pooled experimental conditions that are unshaded.

Table A11 Column 1 shows that suspension gaps and test score gaps each significantly reduced Black parents’ desire to enroll by a similar magnitude of about 0.11. The coefficient for suspension gaps (b=-0.057) means that, on average, parents reviewing a school with a suspension gap reduces parents desires to enroll in Prim by .11 (=-0.057x2) compared to parents who reviewed schools without suspension gaps, regardless of the test score gap. Since the standard deviation for the desire to enroll factor score is approximately 0.88, the effect of the suspension gap is to about 0.129 standard deviations ($0.129=\frac{-0.057\times2}{0.88}$ ). The effect of the test score gap was -0.055, meaning that test score gaps lowered parents’ desires to enroll in Prim by approximately 0.125 standard deviations ($0.125=\frac{-0.055\times2}{0.88}$ ). The insignificant interaction coefficient suggests that the effect of one gap did not differ significantly depending on whether Black parents encountered the other gap. Taken together, the effect coding regression results suggest that Black parents are equally deterred from enrolling in schools with racial disparities in suspensions and racial disparities in students’ academic achievements.

Columns 2 and 3 include regression results for two binary outcomes: the likelihood of enrolling and the likelihood of choosing Prim over the alternative anchor school. The suspension gap reduced Black parents’ likelihood of choosing Prim over Walker by 6.5 percentage points (*b-suspension gap*=-0.0327,p<.01; .065=-0.032x2) relative to not having a suspension gap. However, the suspension gap did not significantly contribute to parents’ likelihood of enrolling in Prim (*b-suspension gap*=-0.0168,p>.10). Columns 2 and 3 also show that the test score gap decreased Black parents’ likelihood of enrolling in Prim by 4.3 percentage points (*b-test score gap* =-0.0217,p<.10) and their likelihood of choosing Prim over the alternative by 4 percentage points (*b-test score gap* =-0.0200,p<.10).

| Table 5: Summary of Walker and Prim School Profile Information | | |
| --- | --- | --- |
| Panel A: Information Held Constant (Not Manipulated with Treatment Status) | | |
| Name | Walker Middle School | Prim Middle School |
| Category | Middle School | Middle School |
| Principal | John Thomas | Carrie Walsh |
| Accreditation | Accredited | Accredited |
| Distance to School | 15-minute walk from your address | 15-minute walk from your address |
| Student-Teacher Ratio | 12 to 1 | 12 to 1 |
| Teachers with Three or More Years of Experience | 68% | 72% |
| Enrollment | Asian: 5% | Asian: 9% |
|  | Black: 42% | Black: 35% |
|  | Hispanic: 20% | Hispanic: 17% |
|  | White: 29% | White: 34% |
|  | Multi-Racial: 4% | Multi-Racial: 5% |
|  | Economically Disadvantaged: 32% | Economically Disadvantaged: 26% |
|  | Students with Disabilities: 13% | Students with Disabilities: 12% |
|  | English Learners: 16% | English Learners: 13% |
| Student Engagement | Chronic Absenteeism: 8% | Chronic Absenteeism: 6% |
| English Pass Rate | 65% | 75% |
| Math Pass Rate | 66% | 75% |

| Panel B: School Test Score and Suspension Rates | | | | | | | | | | | | |
| --- | --- | --- | --- | --- | --- | --- | --- | --- | --- | --- | --- | --- |
|  |  | | Walker  (Not Manipulated with Treatment Status) | | Prim Randomized Levels  (Information in Bold Differed from the No Gap Control Condition) | | | | | | | |
|  |  | |  | | Control:  No Gap | | Treatment 1: Test Score Gap Only | | Treatment 2:  Suspension Gap Only | | Treatment 3:  Both Gaps | |
| English: Percent of Students at Grade Level | Overall | | 65% | | 75% | | 75% | | 75% | | 75% | |
|  | Asian | | 87% | | 93% | | **92%** | | 93% | | **92%** | |
|  | Black | | 65% | | 74% | | **65%** | | 74% | | **65%** | |
|  | Hispanic | | 63% | | 71% | | **67%** | | 71% | | **67%** | |
|  | White | | 65% | | 74% | | **83%** | | 74% | | **83%** | |
|  | Multi-Racial | | 75% | | 82% | | **80%** | | 82% | | **80%** | |
|  | Economically Disadvantaged | | 60% | | 66% | | 66% | | 66% | | 66% | |
|  | English Learner | | 54% | | 63% | | 63% | | 63% | | 63% | |
|  | Students with Disabilities | | 33% | | 41% | | 41% | | 41% | | 41% | |
| Math: Percent of Students at Grade Level | Overall | | 66% | | 75% | | 75% | | 75% | | 75% | |
|  | Asian | | 90% | | 95% | | **97%** | | 95% | | **97%** | |
|  | Black | | 66% | | 74% | | **66%** | | 74% | | **66%** | |
|  | Hispanic | | 68% | | 76% | | **69%** | | 76% | | **69%** | |
|  | White | | 65% | | 74% | | **83%** | | 74% | | **83%** | |
|  | Multi-Racial | | 77% | | 85% | | **78%** | | 85% | | **78%** | |
|  | Economically Disadvantaged | | 62% | | 69% | | 69% | | 69% | | 69% | |
|  | English Learner | | 67% | | 74% | | 74%% | | 74% | | 74% | |
|  | Students with Disabilities | | 39% | | 47% | | 47% | | 47% | | 47% | |
| Suspension Rates | Overall | | 7% | | 4% | | 4% | | 4% | | 4% | |
|  | | Asian | | 3% | | 1% | | 1% | | 1% | | 1% |
|  |  | Black | | 6% | | 3% | | 3% | | **7%** | | **7%** |
|  |  | Hispanic | | 9% | | 5% | | 5% | | **2%** | | **2%** |
|  |  | White | | 9% | | 5% | | 5% | | **3%** | | **3%** |
|  |  | Multi-Racial | | 6% | | 5% | | 5% | | 5% | | 5% |
|  |  | Economically Disadvantaged | | 12% | | 9% | | 9% | | 9% | | 9% |
|  |  | English Learner | | 9% | | 5% | | 5% | | **3%** | | **3%** |
|  |  | Students with Disabilities | | 14% | | 9% | | 9% | | **11%** | | **11%** |

| Table 6: Covariate Balance in the Pooled Sample | | | | | | | | | | |  |
| --- | --- | --- | --- | --- | --- | --- | --- | --- | --- | --- | --- |
|  |  | | Contrasts by Treatment Status | | | | | | |  | |
|  | Control Mean | | Test Score Gap | Suspension Gap | | Both Gaps | | F-statistic | p-value (all = grand mean) | Obs | |
| Male | 0.488*** (0.0244) | | 0.0200 (0.0346) | 0.00658 (0.0345) | | 0.0244 (0.0344) | | 0.219 | 0.883 | 1675 | |
| Black Multiracial/ethnic | 0.0872*** (0.0137) | | 0.0169 (0.0203) | -0.00542 (0.0192) | | -0.0237 (0.0181) | | 1.593 | 0.189 | 1677 | |
| Married | 0.316*** (0.0226) | | 0.00142 (0.0322) | 0.0248 (0.0324) | | 0.0352 (0.0323) | | 0.578 | 0.629 | 1677 | |
| Cohabiting | 0.205*** (0.0197) | | 0.00826 (0.0281) | -0.0125 (0.0275) | | -0.00276 (0.0278) | | 0.191 | 0.903 | 1677 | |
| Less than High School | 0.0660*** (0.0121) | | -0.0249 (0.0155) | -0.00112 (0.0171) | | -0.0164 (0.0161) | | 1.242 | 0.293 | 1677 | |
| High School | 0.304*** (0.0224) | | 0.00779 (0.0319) | -0.0276 (0.0313) | | -0.00209 (0.0316) | | 0.479 | 0.697 | 1677 | |
| Associate's Degree | 0.104*** (0.0148) | | 0.00729 (0.0214) | 0.0140 (0.0217) | | 0.00739 (0.0213) | | 0.140 | 0.936 | 1677 | |
| Some College | 0.257*** (0.0212) | | 0.00847 (0.0304) | 0.00969 (0.0304) | | -0.00870 (0.0299) | | 0.160 | 0.923 | 1677 | |
| BA degree or higher | 0.219*** (0.0201) | | -0.00418 (0.0285) | 0.00429 (0.0287) | | 0.0287 (0.0291) | | 0.501 | 0.682 | 1677 | |
| Previously taught K-12 | 0.111*** (0.0153) | | -0.00673 (0.0214) | -0.0338* (0.0201) | | 0.0118 (0.0221) | | 1.882 | 0.131 | 1677 | |
| Teaches K-12 now | 0.0424*** (0.00979) | | -0.0132 (0.0128) | 0.000908 (0.0140) | | -0.00722 (0.0133) | | 0.546 | 0.651 | 1677 | |
| Age Under 24 | 0.115*** (0.0148) | | -0.0205 (0.0201) | -0.0374* (0.0194) | | -0.0503*** (0.0191) | | 2.584 | 0.0518 | 1677 | |
| Age 25-34 | 0.292*** (0.0221) | | 0.0229 (0.0317) | 0.0518 (0.0322) | | 0.0324 (0.0317) | | 0.900 | 0.440 | 1677 | |
| Age 35-44 | 0.375*** (0.0235) | | -0.0456 (0.0329) | 0.0114 (0.0335) | | -0.00467 (0.0332) | | 1.148 | 0.328 | 1677 | |
| Age 45-54 | 0.158*** (0.0176) | | 0.0525** (0.0265) | -0.0290 (0.0241) | | 0.00475 (0.0249) | | 3.336 | 0.0187 | 1677 | |
| Age 55+ | 0.0591*** (0.0113) | | -0.00932 (0.0157) | 0.00311 (0.0164) | | 0.0178 (0.0171) | | 0.876 | 0.453 | 1677 | |
| Suburban | 0.236*** (0.0207) | | -0.0491* (0.0282) | 0.0505* (0.0303) | | 0.0541* (0.0302) | | 5.698 | 0.000703 | 1677 | |
| Rural | 0.259*** (0.0213) | | 0.0569* (0.0313) | 0.0170 (0.0306) | | -0.00401 (0.0301) | | 1.545 | 0.201 | 1677 | |
| Town | 0.00000610 (0.0000783) | | 0.00477 (0.00338) | -0.0000254 (0.000113) | | 0.00716* (0.00410) | | 1.674 | 0.171 | 1677 | |
| Urban | 0.505*** (0.0243) | | -0.0126 (0.0346) | -0.0675* (0.0344) | | -0.0573* (0.0343) | | 1.841 | 0.138 | 1677 | |
| Northeast | 0.217*** (0.0198) | | -0.0270 (0.0276) | -0.00482 (0.0281) | | 0.0226 (0.0287) | | 1.031 | 0.378 | 1677 | |
| Midwest | 0.264*** (0.0214) | | -0.00399 (0.0303) | 0.00575 (0.0305) | | -0.0413 (0.0294) | | 1.073 | 0.359 | 1677 | |
| South | 0.153*** (0.0175) | | 0.0163 (0.0254) | -0.0185 (0.0242) | | -0.0119 (0.0244) | | 0.740 | 0.528 | 1677 | |
| West | 0.366*** (0.0228) | | 0.0147 (0.0328) | 0.0176 (0.0328) | | 0.0306 (0.0326) | | 0.298 | 0.827 | 1677 | |
| Kids under 18 at Home | 1.955*** (0.0552) | | -0.135* (0.0752) | -0.0748 (0.0826) | | -0.0890 (0.0784) | | 1.093 | 0.351 | 1677 | |
| Notes: Robust standard errors in parentheses. The F-test statistics and p-values for joint significance assess whether the treatment assignment predicts each covariate. | | | | | | | | | | | |
| *** p<0.01, ** p<0.05, * p<0.1 | | |  |  | |  | |  |  |  | |
| Table 7: Sample Comparison to National Benchmarks | | | | | | | | | | |  |
|  | Full Sample | National Benchmark  Estimate | | | Population for the National Benchmark | | Source | | | |  |
|  |  |  | | |  | |  | | | |  |
| Race |  |  | | |  | |  | | | |  |
| Black only | 91.6% | 88.7% | | | Black adults | | Pew Research Center tabulations of the ACS Public Use Microdata Sample, 2022. (Moslimani et al. 2021) | | | |  |
| Black multiracial | 8.4% | 11.3% | | |  |  |  |  |  |  |  |
|  |  |  | | |  | |  | | | |  |
| Marital status | |  | | |  | |  | | | |  |
| Married | 33.2% | 35.5% | | | Parents or householders of Black children under 18 | | NCES tabulations of (ACS), 1-Year Public Use Microdata Sample, 2022. Digest of Education Statistics 2023, table 102.20. (NCES 2023) | | | |  |
|  |  |  | | |  | |  | | | |  |
| Highest level of education | |  | | |  | |  | | | |  |
| BA degree or higher | 22.7% | 32.4% | | | Any related adult in the household of Black children under 18 | | NCES tabulations of (ACS), 1-Year Public Use Microdata Sample, 2022. Digest of Education Statistics 2023, table 104.70. (NCES 2023) | | | |  |
| Associates degree | 11.1% | 12.2% | | |  |  |  |  |  |  |  |
| Some college | 25.9% | 26.3% | | |  |  |  |  |  |  |  |
| High school | 29.9% | 23.6% | | |  |  |  |  |  |  |  |
| Less than high school | 5.5% | 5.5% | | |  |  |  |  |  |  |  |
|  |  |  | | |  | |  | | | |  |
| Locale |  |  | | |  | |  | | | |  |
| Rural | 27.7% | 11.9% | | | Black public school students | | US Department of Education. Common Core of Data. "Public Elementary/Secondary School Universe Survey," 2019-20. (NCES 2021) | | | |  |
| Suburban | 25.0% | 35.8% | | |  |  |  |  |  |  |  |
| Town | 0.3% | 6.9% | | |  |  |  |  |  |  |  |
| Urban | 47.0% | 45.4% | | |  |  |  |  |  |  |  |
|  |  |  | | |  | |  | | | |  |
| Region |  |  | | |  | |  | | | |  |
| Northeast | 20.4% | 16.6% | | | Black adults | | Pew Research Center tabulations of the ACS Public Use Microdata Sample, 2022. (Moslimani et al. 2021) | | | |  |
| Midwest | 25.4% | 17.2% | | |  |  |  |  |  |  |  |
| South | 38.2% | 55.8% | | |  |  |  |  |  |  |  |
| West | 15.0% | 10.3% | | |  |  |  |  |  |  |  |
|  |  |  | | |  | |  | | | |  |
| N | 1677 |  | | |  | |  | | | |  |

| Table 8: Description of Outcomes and Factor Loadings  Panel A: Desire to Enroll Scale | | | | | | |
| --- | --- | --- | --- | --- | --- | --- |
|  |  |  |  |  | Factor Loadings  Desire to Enroll Scale  Estimate (Std. Err) | |
| Name | Question | Response | Mean | SD | Wave 1 | Wave 2 |
| Choose | I would choose Prim Middle School over Walker Middle School. | 1=“Very Unlikely”  2=“Somewhat Unlikely”  3=“Somewhat Likely”  4=“Very Likely” | 2.86 | 0.96 | 0.780 (0.014) | 0.826 (0.011) |
| Enroll | How likely would you be to enroll your child in Prim Middle School? |  | 2.85 | 0.91 | 0.884 (0.008) | 0.859 (0.009) |
| Excited | I would be excited to enroll my child at Prim Middle School. | 1=“Strongly Disagree”  2=“Somewhat Disagree”  3=“Somewhat Agree”  4=“ Strongly Agree” | 2.84 | 0.87 | 0.906 (0.007) | 0.886 (0.008) |
| Fit | Prim Middle School would be a great fit for my child. |  | 2.83 | 0.88 | 0.889 (0.009) | 0.885 (0.008) |
| Disappoint ^a^ | Enrolling my child in Prim Middle School would be disappointing. | 1=“Strongly Disagree”  2=“Somewhat Disagree”  3=“Somewhat Agree”  4=“ Strongly Agree” | 2.73^a^ | 0.97 | 0.647 (0.017) | 0.661 (0.017) |
| Avoid ^a^ | I would do everything realistically in my power to avoid Prim Middle School. |  | 2.76^a^ | 1.01 | 0.679 (0.017) | 0.678 (0.017) |
|  |  |  |  |  | N = 775 | N=901 |

1. We reverse coded the negatively worded items (Disappoint and Avoid) so that lower numbers (e.g., 1 “Strongly Disagree”) represented a high desire to enroll, consistent with the other items.

| Panel B: Expected Belonging Scale | | | | | |
| --- | --- | --- | --- | --- | --- |
|  |  |  |  |  | Factor Loadings  Estimate (Std. Err) |
| Name | Item | Response | Mean | SD | Wave 2 |
| Supported | How likely is it that your student would feel socially and emotionally supported in Prim Middle School? | 1=“Very Unlikely”  2=“Somewhat Unlikely”  3=“Somewhat Likely”  4=“Very Likely” | 2.95 | 0.84 | 0.859 (0.020) |
| Friends | How likely is it that your student would make friends in Prim Middle School? |  | 3.18 | 0.82 | 0.827 (0.021) |
| Welcome | In general, how welcome do you think your student would feel in Prim Middle School? | 1=“Very Unwelcome”  2=“Somewhat Unwelcome”  3=“Somewhat Welcome”  4=“Very Welcome” | 2.60 | 1.10 | 0.693 (0.021) |
|  |  |  |  |  | N = 901 |

*Source:* Hailey and Murray (2025)

Table 9: Effects of Suspension and Test Score Gaps on Desire to Enroll and Choice (Wave 1)

|  | (1) | (2) | (3) |
| --- | --- | --- | --- |
| VARIABLES | Desire to Enroll | P(Enroll at Prim) | P(Choose Prim) |
| No Gap (reference) |  |  |  |
| Suspension Gap | -0.152* | -0.0433 | -0.0897* |
|  | (0.0910) | (0.0473) | (0.0472) |
| Test Score Gap | -0.0470 | -0.0124 | -0.0694 |
|  | (0.0879) | (0.0470) | (0.0476) |
| Suspension and Test Score Gap | -0.250*** | -0.0758 | -0.139*** |
|  | (0.0884) | (0.0471) | (0.0476) |
| Constant | 0.155 | 0.717*** | 0.868*** |
|  | (0.269) | (0.140) | (0.136) |
|  |  |  |  |
| Observations | 774 | 774 | 773 |
| R-squared | 0.044 | 0.025 | 0.035 |

Robust standard errors in parentheses *** p<0.01, ** p<0.05, * p<0.1

Notes: Column 1 shows effects on our factor score for desire to enroll, which is approximately normal. Columns 2 and 3 show effects from linear probability models on the likelihood of enrolling at Prim or choosing Prim over the alternative anchor school. The covariates are an indicator for whether the parent was male, an indicator for identifying as multi-racial in addition to Black, an indicator for having a BA degree or higher, an indicator for being married, and indicator for cohabiting, an indicator for currently or previously being a K-12 teacher, indicators for locale (suburban, town, rural, urban), and age group.

Table 10: Effects of Suspension and Test Score Gaps on Desire to Enroll and Choice (Wave 2)

|  | (1) | (2) | (3) | (4) |
| --- | --- | --- | --- | --- |
| VARIABLES | Desire to Enroll | P(Enroll at Prim) | P(Choose Prim) | Expected Belonging |
| No Gap (reference) |  |  |  |  |
| Suspension Gap | -7.71e-05 | 0.0405 | 0.0112 | -0.0430 |
|  | (0.0820) | (0.0415) | (0.0427) | (0.0874) |
| Test Score Gap | -0.0844 | -0.00849 | 0.0314 | -0.0656 |
|  | (0.0828) | (0.0417) | (0.0414) | (0.0844) |
| Suspension and Test Score Gap | -0.217*** | -0.0860** | -0.0824* | -0.186** |
|  | (0.0839) | (0.0438) | (0.0440) | (0.0879) |
| Constant | -0.0900 | 0.824*** | 0.644*** | -0.00665 |
|  | (0.171) | (0.0883) | (0.0901) | (0.180) |
|  |  |  |  |  |
| Observations | 901 | 901 | 901 | 901 |
| R-squared | 0.050 | 0.040 | 0.046 | 0.036 |

Robust standard errors in parentheses *** p<0.01, ** p<0.05, * p<0.1

Notes: Column 1 shows effects on our factor score for desire to enroll, which is approximately normal. Columns 2 and 3 show effects from linear probability models on the likelihood of enrolling at Prim or choosing Prim over the alternative anchor school. The covariates are an indicator for whether the parent was male, an indicator for identifying as multi-racial in addition to Black, an indicator for having a BA degree or higher, an indicator for being married, and indicator for cohabiting, an indicator for currently or previously being a K-12 teacher, indicators for locale (suburban, town, rural, urban), and age group.

Table 11: Effects of Suspension and Test Score Gaps on Desire to Enroll and Choice (Effect-coded Regression Results)

|  |  | Pooled Sample |  |  |  |
| --- | --- | --- | --- | --- | --- |
|  | (1) | (2) | (3) |  |  |
|  | Desire to enroll | P(Enroll) | P(Choose) |  |  |
|  |  |  |  | N compared | Conditions Compared |
| Suspension gap | -0.0569*** | -0.0168 | -0.0327*** | 839 | (T2:Suspension gap only) |
|  | (0.0215) | (0.0112) | (0.0113) |  | (T4: Both gaps) |
|  |  |  |  | 836 | (Control: No gaps) |
|  |  |  |  |  | (T1: Test score gap only) |
|  |  |  |  |  |  |
| Test score gap | -0.0548** | -0.0217* | -0.0200* | 837 | (Test score gap only) |
|  | (0.0215) | (0.0111) | (0.0113) |  | (T4: Both gaps) |
|  |  |  |  | 838 | (Control: No gaps) |
|  |  |  |  |  | (T2:Suspension gap only) |
|  |  |  |  |  |  |
| Suspension gap x test score gap | -0.0204 | -0.0173 | -0.0127 | 845 | (Control: No gaps) |
|  | (0.0215) | (0.0111) | (0.0112) |  | (T4: Both gaps) |
|  |  |  |  | 830 | (T2:Suspension gap only) |
|  |  |  |  |  | (T1: Test score gap only) |
| Constant | -0.0766 | 0.696*** | 0.703*** |  |  |
|  | (0.142) | (0.131) | (0.122) |  |  |
| Observations | 1,675 | 1,675 | 1,674 |  |  |
| R-squared | 0.033 | 0.023 | 0.028 |  |  |

Robust standard errors in parentheses *** p<0.01, ** p<0.05, * p<0.1

Notes: Column 1 shows results from OLS regressions estimating Equation 2 using effect coding in both waves of data collection with a fixed effect for wave. Column 1 shows effects on our factor score for desire to enroll, which is approximately normal. Columns 2 and 3 show effects from linear probability models on the likelihood of enrolling at Prim or choosing Prim over the alternative anchor school. The covariates are an indicator for whether the parent was male, an indicator for identifying as multi-racial in addition to Black, an indicator for having a BA degree or higher, an indicator for being married, and indicator for cohabiting, an indicator for currently or previously being a K-12 teacher, indicators for locale (suburban, town, rural, urban), and age group. Each effect-coded coefficient comes from approximately splitting the sample in half, so the standard errors are generally consistent within each column.

Table 12: Response-Time Attentiveness Clustering

| Panel A: Wave 1 |  |  |  |
| --- | --- | --- | --- |
| Cluster | Count | Percent | Average Survey Completion Time (Seconds) |
| Fast | 372 | 47.94 | 115.84 |
| Medium | 340 | 43.81 | 326.66 |
| Slow | 64 | 8.25 | 664.22 |
| Panel B: Wave 2 |  |  |  |
| Cluster | Count | Percent | Average Survey Completion Time (Seconds) |
| Fast | 301 | 33.41 | 174.23 |
| Medium | 515 | 57.16 | 479.07 |
| Slow | 85 | 9.43 | 1551.24 |

Notes. Panels A and B show the results of response-time attentiveness clustering based on two response time measures (Read et al., 2021, 2022). The first measure is the number of seconds respondents took to review the page with the school profile but no survey items. The second measure is the number of seconds respondents took to review the page that showed the school profiles alongside the desire to enroll items and (in Wave 2) the expected belonging items. Response-time attentiveness clustering weights response times using principal component analysis and then fits a Gaussian mixture model (GMM) on the weighted response times to assign respondents to attentiveness clusters. We retained two principal components that explained 90% of the total variation across the two response time measures. Response times were analyzed separately by wave since the second wave had more survey items. The third column shows the average total survey completion time by cluster.

**Fig 4** Walker Middle School Profile Held Constant for all Respondents


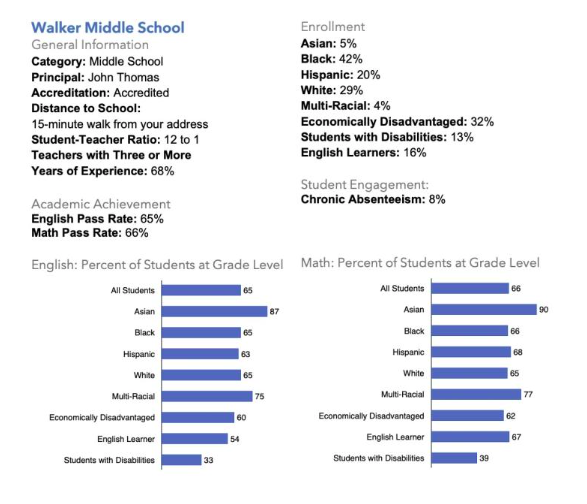


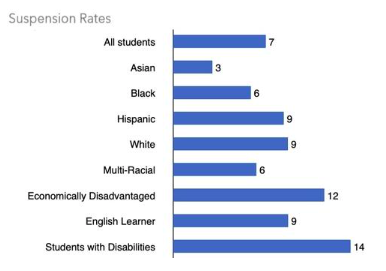


**Fig 5** Typo in Wave 1 that was Corrected in Wave 2


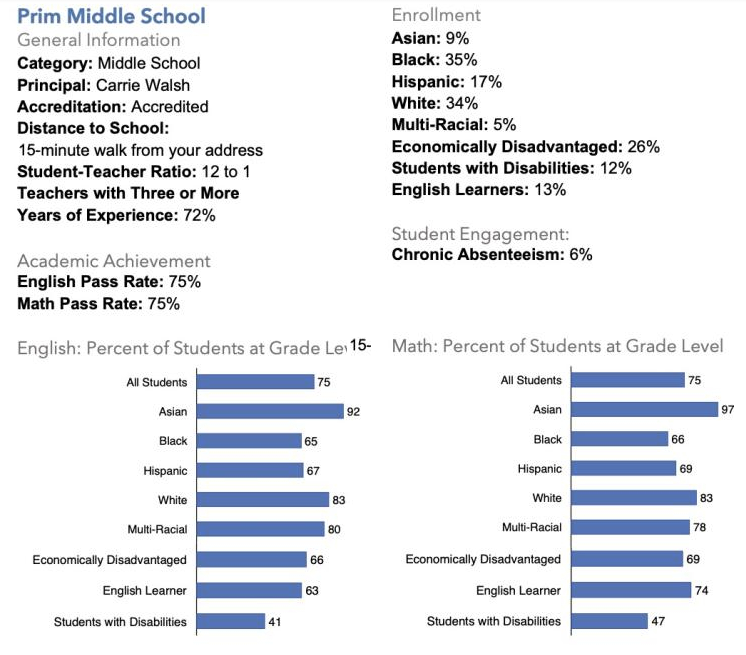

Notes: Figure 6 shows results from OLS regression estimating Equation 1 on desire to enroll stratified by the response-attentiveness clusters described in Table 12. Respondents were categorized into clusters using response-time attentiveness clustering (Read et al., 2021, 2022) separately by wave. Error bars show 95% confidence intervals. Regression covariates include an indicator for whether the parent was male, an indicator for identifying as multi-racial in addition to Black, an indicator for having a BA degree or higher, an indicator for being married, and indicator for cohabiting, an indicator for currently or previously being a K-12 teacher, indicators for locale (suburban, town, rural with urban as the reference), and age, as well as a fixed effect for wave.
